# Impact of the COVID-19 pandemic on migraine in Japan: a multicentre cross-sectional study

**DOI:** 10.1186/s10194-021-01263-1

**Published:** 2021-06-07

**Authors:** Keisuke Suzuki, Takao Takeshima, Hisaka Igarashi, Noboru Imai, Daisuke Danno, Toshimasa Yamamoto, Eiichiro Nagata, Yasuo Haruyama, Takashi Mitsufuji, Shiho Suzuki, Yasuo Ito, Mamoru Shibata, Hisanori Kowa, Shoji Kikui, Tomohiko Shiina, Madoka Okamura, Muneto Tatsumoto, Koichi Hirata

**Affiliations:** 1 Department of Neurology, Dokkyo Medical University, 880 Kitakobayashi, Mibu, Shimotsuga, 321-0293 Tochigi, Japan; 2 Department of Neurology, Headache Center, Tominaga Hospital, Osaka, Japan; 3 Department of Internal Medicine, Headache Care Unit, Fujitsu Clinic, Kanagawa, Japan; 4 Department of Neurology, Japanese Red Cross Shizuoka Hospital, Shizuoka, Japan; 5 Department of Neurology, Saitama Medical University, Saitama, Japan; 6 Department of Neurology, Tokai University School of Medicine, Kanagawa, Japan; 7 Integrated Research Faculty for Advanced Medical Science, Dokkyo Medical University School of Medicine, Tochigi, Japan; 8 Department of Neurology, Tokyo Dental College Ichikawa General Hospital, Chiba, Japan; 9 Department of Neurology, National Hospital Organization, Matsue Medical Center, Shimane, Japan; 10 Medical Safety Management Center, Dokkyo Medical University Hospital, Tochigi, Japan

**Keywords:** Migraine, COVID-19, Headache-related disability, Sleep disturbances, Anxiety, Depression

## Abstract

**Objectives:**

To assess the impacts of social situation changes due to the coronavirus disease 2019 (COVID-19) pandemic on headache-related disability and other symptoms in patients with migraine in Japan.

**Methods:**

We conducted a multicentre, cross-sectional study including 659 outpatients with migraine diagnosed by headache specialists. The participants were asked about the impacts of the first wave of the COVID-19 pandemic on headache-related disability, headache days, headache intensity, stress, physical activity, hospital access and their work and home lives. For headache-related disability, the total Migraine Disability Assessment (MIDAS) score and part A and B scores were analysed. Multivariate stepwise linear regression analysis was performed to identify the clinical predictors of changes in the total MIDAS score before and during the COVID-19 pandemic. Logistic regression analysis was performed to determine the factors related to new-onset headache during the COVID-19 pandemic.

**Results:**

Finally, 606 migraine patients (73 M/533 F; age, 45.2 ± 12.0 years) were included in the study, excluding those with incomplete data. Increased stress, substantial concern about COVID-19 and negative impacts of the first wave of the COVID-19 pandemic on daily life were reported in 56.8 %, 55.1 and 45.0 % of the participants, respectively. The total MIDAS and A and B scores did not significantly change after the first wave of the COVID-19 pandemic. New-onset headache, which was observed in 95 patients (15.7 %), was associated with younger age and worsened mood and sleep in the logistic regression analysis. The multivariate stepwise linear regression analysis of changes in the total MIDAS score before and during the first wave of COVID-19 pandemic identified worsened sleep, increased acute medication use, increased stress, medication shortages, comorbidities, the absence of an aura and new-onset headache were determinants of an increased total MIDAS score during the first wave of the COVID-19 pandemic.

**Conclusions:**

In this multicentre study, clinical factors relevant to headache-related disability, such as new-onset headache, stress and sleep disturbances, were identified, highlighting the importance of symptom management in migraine patients during the first wave of the COVID-19 pandemic.

**Supplementary Information:**

The online version contains supplementary material available at 10.1186/s10194-021-01263-1.

## Introduction

The spread of coronavirus disease 2019 (COVID-19), caused by severe acute respiratory syndrome coronavirus 2 (SARS-CoV-2), has caused a global pandemic since its emergence in Wuhan, China, in December 2019 [1]. In Japan, the infection began to spread in late February 2020, and in April, during the first wave of the COVID-19 pandemic, the first nationwide state of emergency declaration was issued, requesting people to refrain from leaving their homes to prevent the further spread of COVID-19. Although the first state of emergency declaration ended at the end of May 2020, people continued to avoid crowds and refrain from non-essential outings. The Ministry of Health, Labour and Welfare of Japan declared that people should avoid three Cs during the COVID-19 pandemic: (1) closed spaces with poor ventilation; (2) crowded places with many people nearby; and (3) close-contact situations, such as close-range conversations. These recommendations reflect the fact that the risk of cluster occurrence is particularly high when the three Cs overlap [2]. Thus, lifestyles and social situations changed dramatically after activities were restricted to maintain social distancing. Due to this situation, some medical institutions, including our hospitals, introduced telemedicine to prevent the spread of infection.

An online survey of 3,637 COVID-19-free individuals from China in February during the COVID-19 pandemic reported increases in the prevalence of insomnia, anxiety, and depressive symptoms [3]. Similarly, a study from Spain, which investigated the psychological effects of the COVID-19 pandemic in 976 adults, found increases in anxiety, stress, and depression after the nationwide state of alert was issued [4]. This indicates that the impacts of changing social conditions are significant, even in individuals not infected with SARS-CoV-2.

Significant negative impacts on hospital-based headache care and research have been reported during the COVID-19 pandemic in Denmark and Norway [5]. While telemedicine is recommended by the American Headache Society for migraine patients [6], concerns have been raised about the impacts of the discontinuation of outpatient behavioural therapy for chronic migraine, headache medication overuse in the outpatient setting [7] and the discontinuation of botulinum A toxin and occipital nerve block therapy for intractable headache [8]. Stress and post-stress rest are known migraine triggers [9, 10], and migraine is associated with various psychiatric comorbidities [11]. Patients with migraine have double the risk of developing post-traumatic stress disorder after trauma [12], and symptoms of post-traumatic stress disorder are associated with higher odds of experiencing frequent migraines after a natural disaster [13]. Therefore, it is likely that increased stress during the COVID-19 pandemic affects migraine.

However, the impacts of changes in social situations during the COVID-19 pandemic on migraine have not yet been addressed. We designed a multicentre, cross-sectional study to investigate the effects of the first wave of the COVID-19 pandemic on headache-related disability and other clinical symptoms in migraine patients in Japan.

## Methods

### Study design and setting

**Fig. 1:**
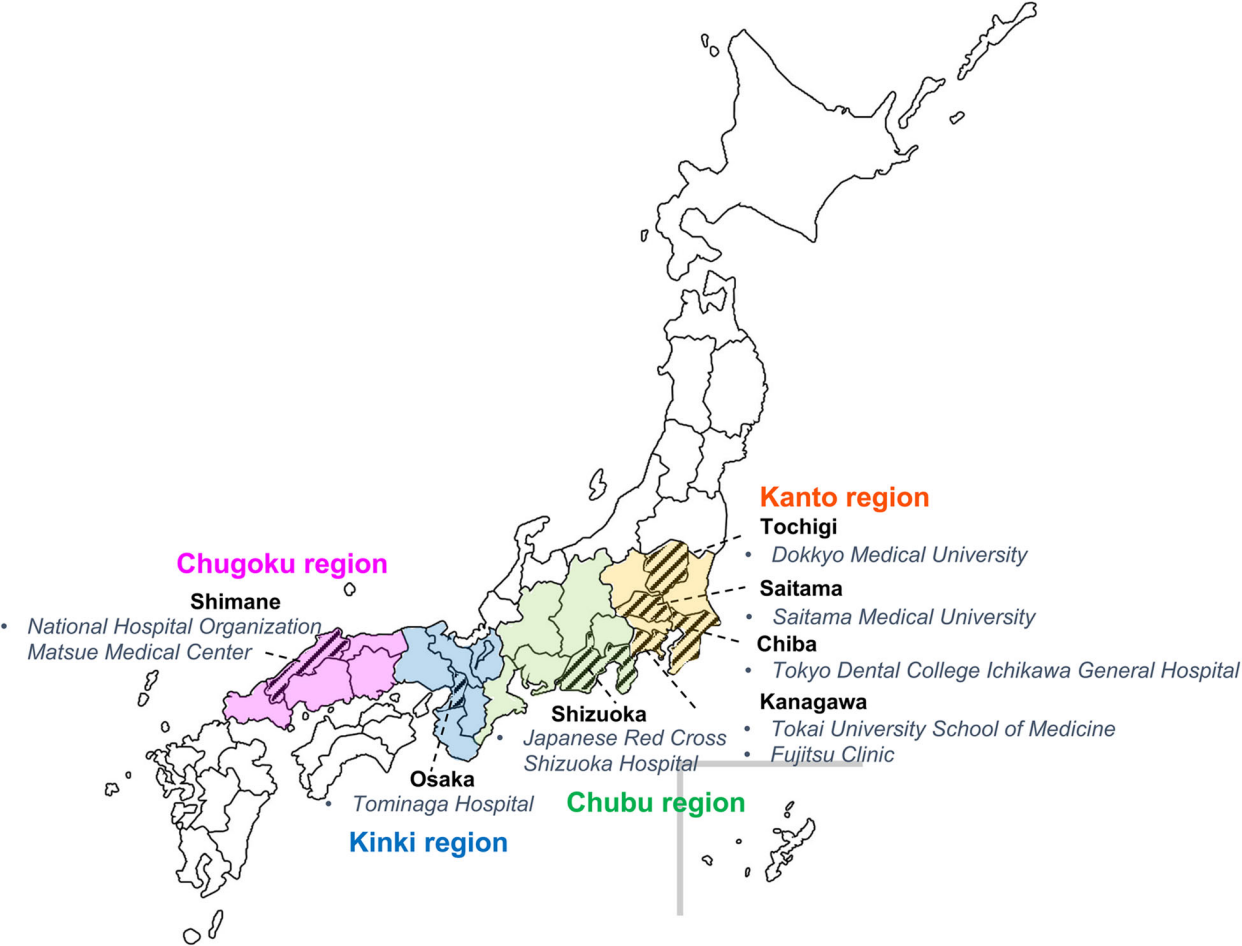
Headache centres participated in this study

This multicentre cross-sectional study was conducted from June to December 2020 in accordance with the Strengthening the Reporting of Observational Studies in Epidemiology (STROBE) Statement and the principles of the Declaration of Helsinki and was approved by the institutional review boards of the participating facilities. Figure 1 shows the data pertaining to the 8 headache centres that participated in this study. Each facility serves as a representative centre for four regions, namely, the Kanto, Chubu, Kinki and Chugoku regions, in Japan.

### Participants

Consecutive outpatients with migraine were recruited from multiple centres. The diagnosis of episodic or chronic migraine was made by headache specialists according to the International Classification of Headache Disorders, 3rd edition [14]. Patients were also classified as having migraine with or without aura. All patients underwent head imaging to rule out secondary headache and had been treated for migraine for at least several months. Patients with additional headache disorders were excluded, except for those with tension-type headache. Based on the mean (14.7) and SD (18.7) of the total Migraine Disability Assessment (MIDAS) score in 161 migraine patients [15], the difference in the MIDAS score due to the impact of the COVID-19 pandemic was assumed to be 2.0. We computed the required sample size (two-tailed, effect size 0.12, alpha 0.05, power 0.8) for the Wilcoxon signed-rank test using G*Power software (version 3.3.9.7) [16]. The sample size needed was 577. Considering missing responses, we planned to recruit 635 patients. The exclusion criteria were as follows: patients with organic brain disease other than migraine, patients with cognitive impairment, patients with incomplete data for the MIDAS score and patients who were being treated or had been treated for COVID-19. All participants provided written informed consent to participate in the study. For patients under 18 years of age, written informed consent was also obtained from their parents.

### Clinical assessments

Based on clinical medical records and headache diaries, the following information was obtained: patient age, sex, detailed features of migraine, duration of illness, habits (smoking, alcohol consumption, and caffeine consumption), and acute and chronic medications for migraine at the time of the study. Accompanying symptoms, the presence of photo-/phono-/osmophobia, and the presence of allodynia were assessed by clinical examinations and interviews performed by neurologists. The questionnaire used in this study is shown in supplementary Table S1. The participants were asked about the impacts of the first wave of the COVID-19 pandemic on stress, physical activity and their work and home lives (frontline worker, switching from working outside the home to working at home, and occupation) and if they had received prescriptions for medications via a telephone or online consultation. Participants’ interest in and concern about COVID-19 and new-onset headache during the first wave of the COVID-19 pandemic were assessed. New-onset headache was defined as being present if the patients reported that they had developed a headache that was different in nature and intensity from their usual migraines after the declaration of the state of emergency. For those who used a mask, a face guard or personal protective equipment (PPE) (combined use of a surgical mask and a face guard), the duration of daily use was also assessed. The total MIDAS score and part A and B scores [17] were used to compare the degree of disability experienced in daily life related to the presence of headache, headache days and headache intensity during the 3 months before and after the first declaration of the state of emergency in April 2020. Changes in mood and sleep quality during the first wave of the COVID-19 pandemic were assessed by the 7-point patient’s global impression of change (PGIC) scale (1 very much improved to 7 very much worse) [18]. The primary outcomes were the change in the total MIDAS score during the COVID-19 pandemic and the clinical factors, including habits, aura status, sleep, mood, hypersensitivity, and accompanying symptoms, that were predictive of a change in the total MIDAS score.

### Statistical analysis

We annotated each table and analysis with regard to individual missing values. To compare patients with and without new-onset headache, the Mann-Whitney U test or Student’s t test was used for continuous variables, as appropriate, and the chi-square test was employed for categorical variables. The Wilcoxon signed-rank test was used to compare the total MIDAS score and part A and B scores before and during the COVID-19 pandemic, as the differences between pairs of data were non-normally distributed according to the Kolmogorov-Smirnov test. Multivariate stepwise linear regression analysis was performed to identify the clinical factors that were predictive of changes in the total MIDAS score before and during the COVID-19 pandemic. Logistic regression analysis using likelihood ratio forward selection was performed to determine the factors related to new-onset headache during the COVID-19 pandemic. Two-tailed *p* values < 0.05 were considered to be statistically significant. IBM SPSS Statistics V.26.0 (IBM SPSS, Tokyo, Japan) was used for the statistical analyses. GraphPad Prism for Mac (V.8.43; GraphPad Software, San Diego, California, USA) was used to generate the figures.

## Results

### Characteristics of migraine patients during the COVID-19 pandemic

**Fig. 2:**
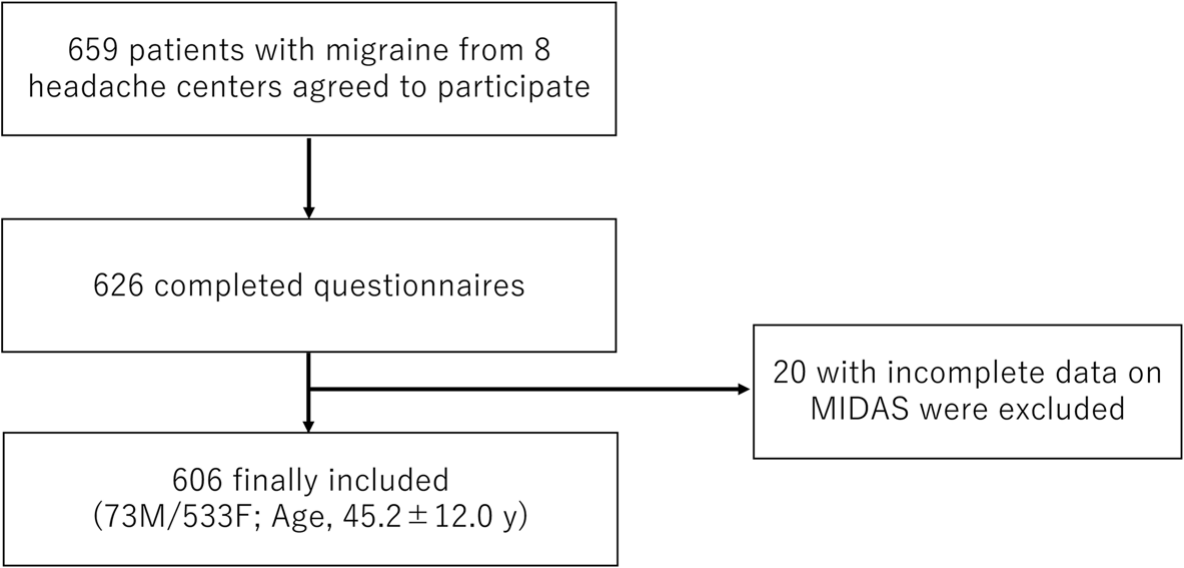
Study flowchart

Among the 659 initially recruited migraine patients, 626 completed the questionnaires and 606 (73 M/533 F: age, mean 45.2 ± 12.0 years; range 11–77 years) were finally included in the study; 20 patients with incomplete data for the MIDAS score were excluded (Fig. 2). Six of the 606 patients (1 %) were under the age of 18 years. Seven patients (1.2 %) had comorbid tension-type headache. Table 1 shows the demographics and characteristics of patients with migraine in this study. A total of 98.3 and 63.4 % received acute and preventive treatments for migraine, respectively. The details are shown in Table S2. In our study, 99.5 % used infection protection devices: 99 % used a mask, 6.1 % used a face shield, and 1.7 % used PPE. Increased stress, great concerns about COVID-19 and negative impacts of COVID-19 pandemic on daily life were reported in 56.8 %, 55.1 and 45.0 % of the participants, respectively (Table 2). Dose or type of acute headache treatment increased by 30.7 %, and preventive headache treatment was newly prescribed or increased by 15.5 %.

**Table 1:** Demographics and clinical characteristics of patients with migraine

	Patients with migraine
n (M/F)	606 (73/533)
Age, years	45.2 ± 12.0
Diagnosis, n (%)	
Migraine without aura	483 (79.7)
Migraine with aura	139 (22.9)
Chronic migraine	84 (13.9)
Onset of migraine^a^, years	20.2 ± 10.2
Smoking, n (%)	
Never	457 (75.4)
Past	103 (17.0)
Current	46 (7.6)
Alcohol intake^a^, n (%)	
Never	291 (48.0)
<1 day/week	223 (36.8)
1–2 days/week	60 (9.9)
3–5 days/week	17 (2.8)
6–7 days/week	13 (2.1)
Caffeine, n (%)	560 (92.4)
Caffeine^a^, cups/day	2.7 ± 2.1
Accompanying symptoms, n (%)	
Nausea	424 (70.0)
Photophobia	439 (72.4)
Phonophobia	438 (72.3)
Osmophobia	312 (51.5)
Allodynia	102 (16.8)
^b^Acute headache medication, n (%)	596 (98.3)
^b^Preventive headache medication, n (%)	384 (63.4)
Comorbidities, n (%)	269 (44.4)
Use of infection protective wear, n (%)	603 (99.5)
Mask, n (%)	603 (99.5)
Mask usage^a^ (h/d)	6.7 ± 4.0
Face shield, n (%)	37 (6.1)
Face shield usage^a^ (h/d)	4.6 ± 3.1
PPE, n (%)	10 (1.7)
PPE usage (h/d)	4.0 ± 3.1
Evaluation period after the end of the state of emergency (months)	2.6 ± 1.8

*PPE *personal protective equipment

^a^Missing values (onset of migraine = 6, alcohol intake = 2, caffeine = 11, mask time = 22, and face shield time = 1) were excluded

^b^At the time of the study

**Table 2:** Impact of the COVID-19 pandemic on clinical symptoms, hospital visits and medication usage

	Migraine patients (*n* = 606)
Stress, n (%)	
Decreased	30 (5.0)
Unchanged	232 (38.3)
Increased	344 (56.8)
Physical activity, n (%)	
Decreased	305 (50.3)
Unchanged	268 (44.2)
Increased	33 (5.4)
Impact of COVID-19 epidemic on daily life, n (%)	273 (45.0)
Interest in COVID-19, n (%)	
None	1 (0.2)
Very little	3 (0.5)
Little	37 (6.1)
Moderate	139 (22.9)
Strong	426 (70.3)
Concerns about COVID-19, n (%)	
None	4 (0.7)
Very minor	10 (1.7)
Minor	83 (13.7)
Moderate	175 (28.9)
Great	334 (55.1)
PGIC scale (1–7)	
Mood	4.5 ± 0.9
Sleep	4.4 ± 0.99
^a^Acute headache treatment, n (%)	
Decreased	53 (8.7)
Unchanged	367 (60.6)
Increased	186 (30.7)
Addition or changes in headache-prevention medications, n (%)	94 (15.5)
Occupation, n (%)	
Frontline worker	193 (31.8)
Working from home	224 (37.0)
Other occupations	189 (31.2)
Problems with hospital access, n (%)	95 (15.7)
Medication shortage, (n%)	43 (7.1)
Received online medical care	
Yes, n (%)	95 (15.7)
- If yes, what are the advantages and disadvantages?	
Advantages	
Infection risk reduction	91 (95.8)
Able to continue working	22 (23.2)
Remain with children	10 (10.5)
Others	13 (13.7)
Disadvantages	
Short examination times	35 (36.8)
Time schedule conflicts	4 (4.2)
Unfamiliar with the method	4 (4.2)
None	48 (50.5)
Others	5 (5.3)
No, n (%)	511 (95)
- If no, would you like to receive telephone/online care in the future?	264 (51.7)

*PGIC *patient global impression of change

^a^dose or type, after the state of emergency declaration

### Impact of the COVID-19 pandemic on hospital visits and medications

Problems accessing the hospital were reported by 15.7 % of the participants, and a headache medication shortage was reported by only 7.1 % of the participants. During the COVID-19 pandemic, 15.7 % of the participants received online medical care; the advantages included infection risk reduction, which was reported by the majority (95.8 %) of patients, and the ability to continue working, which was reported by 23.2 %. The disadvantages included short examination times, as reported by 36.9 %.

### Characteristics and determinants of new-onset headache

New-onset headache was observed in 95 (15.7 %) patients (Table 3). The headache presentation was variable. There were wide overlaps in the region and presentations of new-onset headaches, with 65.3 % being pulsatile headaches and 64.2 % being pressing headaches. Patients with new-onset headache had a higher rate of aura, greater impact of COVID-19 on their daily lives, higher rate of medication shortage, greater concerns about COVID-19, higher stress levels, worsened mood and sleep, and a higher rate of mask usage (h/d) than patients without new-onset headache. Additionally, the total MIDAS and A and B scores were significantly higher in those with new-onset headache than in those without new-onset headache during the pandemic (Table S3). A logistic regression analysis using likelihood ratio forward selection showed that younger age and worsened mood and sleep contributed to new-onset headache (Table S4).

**Table 3:** Characteristics of new-onset headache after the COVID-19 pandemic (*n* = 95)

	New-onset headache (*n* = 95)
Headache region, n (%)	
Orbital	49 (51.6)
Frontal	29 (30.5)
Temporal	54 (56.8)
Posterior	42 (44.2)
Entire	13 (13.7)
Others	9 (9.5)
Lateralization, n (%)	
Unilateral	56 (58.9)
Bilateral	43 (45.3)
Headache duration, n (%)	
Less than 4 h	47 (49.5)
4 to 72 h	29 (30.5)
4 days or longer	19 (20.0)
Headache presentations, n (%)	
Pulsatile	62 (65.3)
Pressing	61 (64.2)
Sharp	15 (15.8)
Thunderclap	5 (5.3)
Headache intensity, n (%)	
Mild	20 (21.1)
Moderate	47 (49.5)
Strong	28 (29.5)
Headache days per month	8.7 ± 7.9

### Changes in and determinants of the MIDAS score

**Fig. 3:**
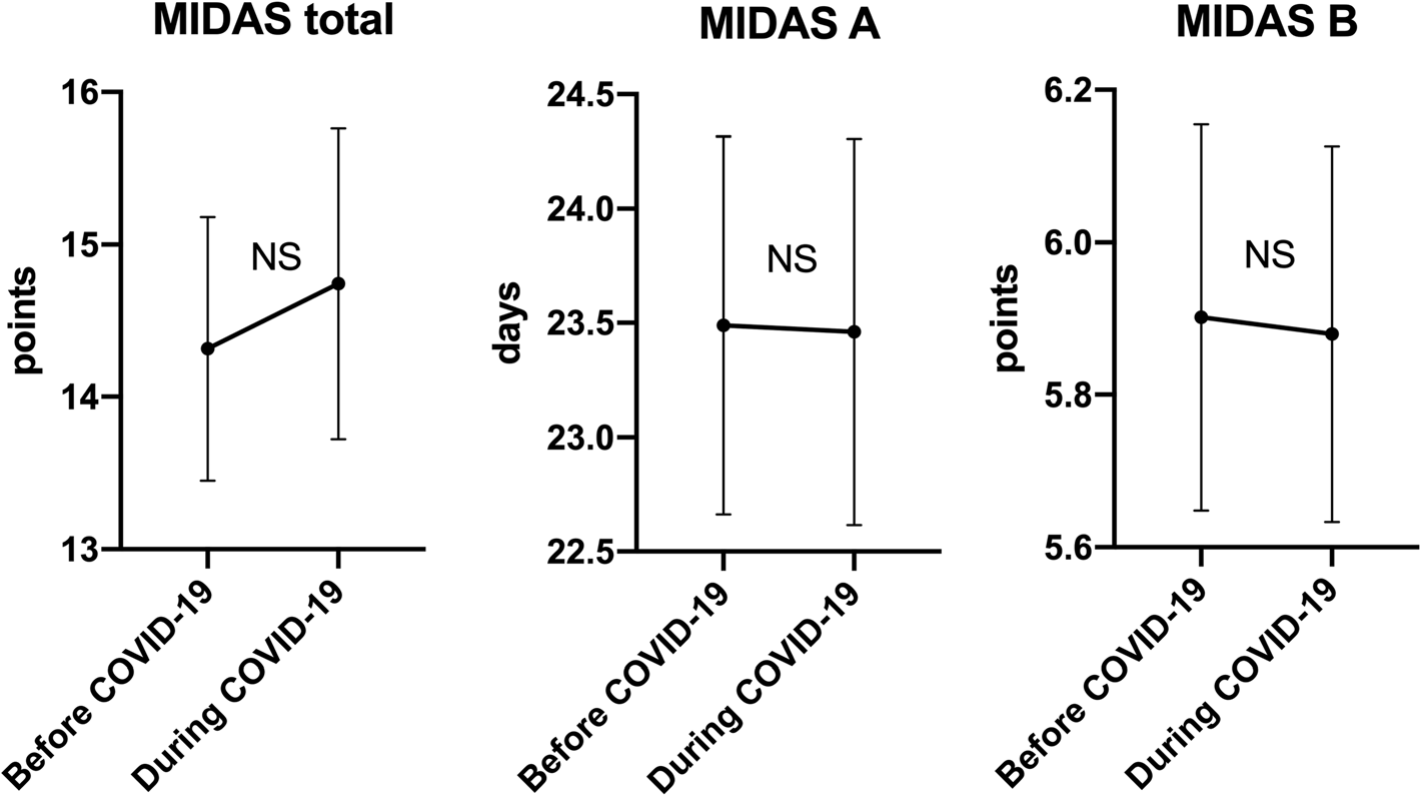
Changes in MIDAS total, A and B scores before and during the COVID-19 pandemic. NS = not significant. Error bars represent standard errors of the mean. The total MIDAS (14.3 ± 0.9 vs. 14.7 ± 1.0, *p* = 0.495) and part A (23.5 ± 0.8 vs. 23.5 ± 0.9, *p* = 0.277) and part B (5.9 ± 0.3 vs. 5.9 ± 0.3, *p* = 0.555) scores were not significantly different before and during the COVID-19 pandemic according to the Wilcoxon signed rank test

Out of the 606 patients, 446 patients (73.6 %) reported their headache days on the MIDAS based on their headache diaries. Overall, the total MIDAS score and part A and B scores were not significantly different before and during the COVID-19 pandemic (Fig. 3). A stepwise linear regression analysis of changes in the total MIDAS score identified sleep disturbances, increased acute medication use, increased stress, medication shortage, the presence of comorbidities, the absence of aura and new-onset headache as determinants of an increased total MIDAS score during the COVID-19 pandemic (Table 4).

**Table 4:** Stepwise linear regression analysis of changes in the total MIDAS score before and during the COVID-19 pandemic (*n* = 603)

Variables	Standardized regression coefficient	SE	*P* value	95 % CI
Sleep	2.915	1.033	0.005	0.886 to 4.493
Acute medication use	5.163	1.691	0.002	1.843 to 8.484
Stress	5.422	1.675	0.001	2.132 to 8.712
Medication shortage	11.075	3.653	0.003	3.900 to 18.249
Comorbidities	4.125	1.887	0.029	0.420 to 7.830
Presence of aura	-4.937	2.216	0.026	-9.289 to -0.586
New-onset headache	6.056	2.724	0.027	0.706 to 11.406

The differences in the total MIDAS score were calculated by subtracting the pre-pandemic score from the score during the COVID-19 pandemic. Independent variables included age, sex, smoking, caffeine consumption, alcohol consumption, migraine with or without aura, chronic migraine, nausea, photophobia, phonophobia, osmophobia, allodynia, occupation type, use of personal protective equipment, impact of COVID-19 on daily life, interest in COVID-19, concerns about COVID-19, new-onset headache, comorbidities, changes in stress, mood, sleep quality, physical activity, acute or preventive medication use, problems with hospital access, medication shortage, online medical care and evaluation period after the end of the state of emergency

## Discussion

In this multicentre study, we studied the effects of social distancing during the first wave of the COVID-19 pandemic on headache-related disability, daily life and other various clinical symptoms using clinical information, headache diaries, and questionnaires at multiple headache centres. We found that a significant number of patients reported increased stress, a negative impact of the first wave of the COVID-19 pandemic on their daily lives, concerns about COVID-19 and changes in mood and sleep. A higher migraine risk in healthcare workers than in the general population was reported in a nationwide population-based cohort study [19]. In our study, 98.3 % of the participants were on acute medication, and 63.4 % were on preventive medication; 30.7 % reported increased acute headache treatment, and 15.5 % reported additions to or changes in headache-prevention medication.

Based on the total MIDAS and A and B scores in this study, headache-related disability, the number of headache days and headache intensity did not change during the first wave of the COVID-19 pandemic. This result was contrary to the research hypothesis that increased stress worsens headache and negatively impacts headache-related disability in patients with migraine. In a web survey of 1,018 patients with migraine during the lockdown period, 60 % reported an increase in migraine frequency, 16 % reported a decrease in migraine frequency and 10 % reported progression to chronic migraine. Migraine severity was increased in 64 % of patients [20]. An observational cross-sectional study from Italy including 433 migraine patients found that migraine frequency and intensity were significantly reduced during quarantine compared to the pre-quarantine period and were correlated with an increased number of days at home [21]. In contrast, similar to the results of our study, Verhagen et al. [22] performed a study in 592 migraine patients who used headache e-diaries and showed that the number of migraine days did not change, the daily use of acute medication decreased and well-being scores improved after lockdown due to the COVID-19 outbreak. We agree with speculation made by Verhagen et al. [22] that the combined effects of working from home, reductions in demanding social lives, and the freedom to choose how to organise one’s time contributed to the lack of change in headache status during the COVID-19 pandemic. However, we could not determine the factors that contributed to the lack of a significant change in the MIDAS score before and during the first wave of the COVID-19 pandemic in this study.

In our study, we found that 56.8 % of the patients with migraine reported increased stress, and increased stress was one of determinants of the development of new-onset headaches during the first wave of the COVID-19 pandemic. In a study from China performed during the COVID-19 outbreak, patients with migraine had significantly higher levels of stress than the controls [23]. In a 90-day prospective daily-diary cohort study involving adults with episodic migraine, increased levels of stress were associated with the risk of migraine the next day [24]. During the COVID-19 pandemic, perceived stress was more strongly associated with brooding and COVID-related rumination among patients with migraine than healthy controls [25]. Also, perceived stress has been found to be associated with chronic migraine, depression and anxiety [26]. The utilization of coping strategies to manage stressful life events has been reported to have a substantial impact on migraines in social situations and at work in patients with migraine without aura [27]. Therefore, differences in the strategies for coping with stress during periods of social restrictions, limited access to hospitals and medication shortages may be associated with the number of headache days and nature of headaches in various studies. In our study, we did not assess how the patients coped with their stress; however, low proportions of participants had difficulty accessing a hospital (15.7 %) and reported a medication shortage (7.1 %), which could be factors that would increase stress.

According to the stepwise linear regression analysis, among the many clinical and social characteristics of patients with migraine, the worsening of sleep, increased use of acute medications, increased stress, medication shortages, the presence of comorbidities, the absence of aura and new-onset headache were significant contributors to increased headache-related disability during the first wave of the COVID-19 pandemic. During the COVID-19 pandemic, patients with migraine were more likely than patients with other neurological conditions to report worsened anxiety and sleep problems [28]. Other studies addressed changes in headache intensity or migraine days during the COVID-19 pandemic [22], but changes in disability related to headache in patients with migraine have not been well studied.

Other noteworthy findings in our study include the fact that 15.7 % of the patients developed new-onset headache during the pandemic, the nature of which differed from that of their pre-existing migraine headaches. In a study of 158 healthcare workers (64.6 % nurses, 32.3 % physicians, and 3.2 % paramedical staff), pre-existing primary headache was present in 29.1 %, and 81 % had complaints of headache related to wearing PPE. New PPE-associated headaches were associated with the presence of pre-existing primary headaches and combined PPE usage > 4 h/day [29]. Additionally, a study consisting of 383 Italian healthcare providers found that 44 (26.5 %) developed de novo facemask-associated headache [30]. In our study, 31.8 % were involved in frontline work, and 12.5 % were healthcare providers. The increased duration of mask usage (h/d) was significantly longer in patients with new-onset headache than in patients without new-onset headache; however, in the logistic regression analysis, it did not remain a significant factor for new-onset headache. Worsened sleep and mood significantly contributed to new-onset headache in our study after adjustment for clinical factors. However, since the location and presentation of the new headaches were diverse and overlapped, we could not determine their characteristics. In healthcare workers, headaches related to PPE use were associated with photophobia, phonophobia and nausea [29]; however, these factors were not determinants of new-onset headache in our study.

In this study, we asked patients to complete a MIDAS assessment of their condition reflecting the 3-month period before and after the first state of emergency declaration. One limitation is that the state of emergency declaration issued between April and May 2020 was a recommendation, with no enforcement until December, the period of the research. Thus, social distancing in our study setting was based on the premise of voluntary cooperation by the population. Second, although the study period was limited to 7 months and we adjusted for the evaluation period after the end of the first declaration of the state of emergency in the linear regression analysis predicting changes in the total MIDAS score, our study was subject to recall bias. Third, 73.6 % of the patients who kept a headache diary reported the number of headache days on the MIDAS based on their diary. However, the number of days reported on the MIDAS in the remaining patients was self-reported. Fourth, we did not include healthy controls in our study because we intended to focus on changes in clinical symptoms in patients with migraine. Fifth, we identified new-onset headache in several patients; however, the presence of photo/phono/osmophobia, nausea, vomiting, symptom worsening due to physical activity, cranial autonomic symptoms and relief after using triptans were not assessed. The relationships between new-onset headache and wearing a mask and the type of mask worn were unclear. Therefore the association between mask-related headache and new-onset headache was not investigated. Sixth, the PGIC scale is not specifically designed to assess changes in mood or sleep, so the use of this assessment method may have influenced the results of this study. Seventh, we did not have baseline data for our participants in this study, and all the data were collected at the same assessment. Last, in our multicentre study, 83.5 % of the patients received triptans, suggesting a possible selection bias towards migraine patients treated by headache specialists at headache centres. In addition, our study was conducted at multiple major headache centres, but it may not be representative of all migraine patients in Japan.

In conclusion, our study identified several clinical factors contributing to headache-related disability and helped clarify the changes in clinical symptoms in migraine patients during the COVID-19 pandemic, which may contribute to improving the future management of migraine patients. In patients with migraine, new-onset headache, stress and sleep disturbances should be carefully monitored and adequately addressed during the COVID-19 pandemic. Our study is also clinically significant because it reveals the impact of the social conditions associated with COVID-19 infection control measures on migraine, a major neurological disease.

## Supplementary Information

**Additional file 1: Table S1. **Questionnaire. **Table S2. **Acute and preventive treatments for headaches in patients with migraine. **Table S3. **Comparisons between patients with and without new-onset headaches after the COVID-19 pandemic. **Table S4. **Logistic regression analysis results of new-onset headache (*n*=603)

## Data Availability

The relevant data are contained within the paper and supplementary materials. However, the datasets from this study are available from the corresponding author upon reasonable request.

## Abbreviations

COVID-19: Coronavirus disease 2019
MIDAS: Migraine Disability Assessment
PGIC: Patient’s global impression of change
PPE: Personal protective equipment
