# Supplementary material for: Impact of the COVID-19 pandemic on migraine in Japan: a multicentre cross-sectional study
Source: J Headache Pain. 2021 Jun 7;22(1):53. doi: 10.1186/s10194-021-01263-1 (PMC8182734; doi:10.1186/s10194-021-01263-1)
Supplement: Supplementary file 1 — Additional file 1: Table S1. Questionnaire. Table S2. Acute and preventive treatments for headaches in patients with migraine. Table S3. Comparisons between patients with and without new-onset headaches after the COVID-19 pandemic. Table S4. Logistic regression analysis results of new-onset headache (n=603) [file 10194_2021_1263_MOESM1_ESM.docx]

**Supplementary Material**

**Table S1: Questionnaire**

In Japan, in order to prevent the spread of COVID-19, people have refrained from going out and maintained social distance, and our lives have changed. We are investigating the impact of these changes in social conditions on patients with migraine. We would appreciate your cooperation. Please answer all the questions accurately.

Age：　 　　(y), Birth date:

Sex：　□ Male　□ Female

- Do you suffer from any other illnesses besides migraine?

□Yes　 □ No　(disease name:　　　　　　 　　　　　　　　　　　　)

- Habits

Do you smoke?

□　Not at all.

□　I used to smoke.

□　I currently smoke.

Do you drink any caffeinated beverages (coffee, tea, Japanese tea, etc.)?

□　No

□　Yes.　 Drink ( ) cups per day

Do you drink alcoholic beverages?

□　Never　□ <1 day/week　□ 1-2 days/week　□ 3-5 days/week

□ 6-7 days/week

- Impact of COVID-19 on work

Choose one of the following three and the relevant details.

1 □ Continuing work as a frontline worker under the declared state of emergency.

□ Healthcare provider □ Store staff □ Delivery business

□ Other (　　　　　　 　　)

2 □ After the declaration of the state of emergency, I have refrained from going out and have been working at home.

　□ Housekeeper □ Office worker □ Public officer □ Teaching staff

□ Service industry □ Other (　　　　　 　　 　)

3 □ Other occupation (　　　　　　　 　 )

- Do you wear a mask or face guard in your daily life to prevent infection?

□Yes　□No

If yes, please list all of the following types and average hours/day you wear them.

□ Mask　　　　 　 (　　 ) hours per day

□ Face guards/goggles (　　　) hours per day

□ Personal Protective Equipment (PPE) (　　　) hours per day

□ Others　　　　 ( ) hours per day

- Has there been any impact of COVID-19 on your household?

□ No

□ Yes →Please select all that apply from the following.

1 □ Have a child at home due to the suspension of school

2 □ Spouse working from home

3 □ Provision of care for parents at home because of the inability to use day care services or nursing home services

4 □ Reduced income

5 □ Others (　　　 　　　　 )

- Do you have an interest in COVID-19?

□ 1 None　□ 2 Very little　□ 3 Little　□ 4 Moderate　□ 5 Strong

- Do you have any concerns about COVID-19 (including any kind of anxiety or worry related to COVID-19)?

□ 1 None　□ 2 Very little　□ 3 Little　□ 4 Moderate　□ 5 Strong

**Compare the situation after the declaration of the state of emergency with the situation before and answer the questions below.**

- Have you developed a new headache that is totally different in nature and intensity from your regular migraine?

□ Yes　　□ No

If yes, please answer the following questions (1) to (6).

(1) How long does the new headache last?

□　Less than 4 hours □　4 to 72 hours (3 days) □ 4 days or longer

(2) How often do you have them?

( ) times per month

( ) days per month

(3) Where do the headaches occur? (Multiple answers acceptable)

□　Behind the eyes　□　Around the forehead (the front of the head)

□　Temporal (side of the head)　□　Posterior (back of the head)

□　Others (　　　　　　　　 　 )

(4) Does the headache occur unilaterally, bilaterally, or both?

□　Unilateral 　□　Bilateral

(5) Please describe the nature of the headache.

□　Pulsatile

□　Pressing

□　Sharp

□　Thunderclap

(6) Please describe the intensity of the headache (choose one).

□　Unbearably strong pain

□　Moderate pain that interferes with work or schoolwork

□　Mild pain that can be tolerated

- Did you have problems accessing medical facilities?

□ Yes　　□ No

- Have you ever run out of medication because you were unable to see a doctor?

□ Yes　　□ No

- Have you received a prescription for medication through a telephone visit or online consultation without going to a medical facility?

□ Yes→(1)，　□ No→(2)

(1) If “yes”:

- - What were the advantages (multiple answers acceptable)?

□ Reduced risk of infection　□ Ability to continue working　□ Ability to remain with children

□ Others (　　　　　　　　　　　　　　　　　　　　　　　)

- - What were the disadvantages (multiple answers acceptable)?

□ Short examination times □ The time schedule doesn't match up well with my schedule　□ Unfamiliar with the method

□ Others (　　　　　　　　　　　　　　　　　　　　　　)

(2) If “No”

Would you like to participate in telephone visits or online consultations in the future?

□ Yes　　□ No

- Has your stress changed from before?

□ Decreased　□ Unchanged　□ Increased

- Has your physical activity changed from before?

□ Decreased　□ Unchanged　□ Increased

- Has your amount of smoking changed? (Only if you smoke)

□ Decreased　□ Unchanged　□ Increased

- Has the amount of alcohol you drink changed?　(Only if you drink)

□ Decreased　□ Unchanged　□ Increased

- Has your caffeine intake changed? (Only if you take it)

□ Decreased　□ Unchanged　□ Increased

- Has your frequency of the use of acute medications for headache attacks changed?

□ Decreased　□ Unchanged　□ Increased

- Have your preventive medications for headaches been added to or changed?

□ Yes　　□ No

- How often do you have headaches and take acute medication for headaches?

|  | Before | After the declaration of the state of emergency |
| --- | --- | --- |
| Frequency of headache attacks | (　　 　) times/month  (　　　 ) days/month | (　　 　) times/month  (　　　 ) days/month |
| Frequency of acute medication use | (　　 　) times/month  (　　　 ) days/month | (　　 　) times/month  (　　　 ) days/month |

For the following three questions, please circle one of the following seven levels to describe your condition after the declaration of the state of emergency.

1　How is the intensity of the headache?

| 1 | 2 | 3 | 4 | 5 | 6 | 7 |
| --- | --- | --- | --- | --- | --- | --- |
| Very much improved | Much improved | Minimally improved | No change | Minimally worse | Much worse | Very much worse |

2　How is your mood?

| 1 | 2 | 3 | 4 | 5 | 6 | 7 |
| --- | --- | --- | --- | --- | --- | --- |
| Very much improved | Much improved | Minimally improved | No change | Minimally worse | Much worse | Very much worse |

3　How is your sleep?

| 1 | 2 | 3 | 4 | 5 | 6 | 7 |
| --- | --- | --- | --- | --- | --- | --- |
| Very much improved | Much improved | Minimally improved | No change | Minimally worse | Much worse | Very much worse |

**Table S2: Acute and preventive treatments for headaches in patients with migraine**

| **Headache treatment** | **Migraine patients (n=606)** |
| --- | --- |
| Acute treatment, n (%) | 596 (98.3) |
| Non-steroidal anti-inflammatory drugs | 370 (61.1) |
| Triptans | 506 (83.5) |
| Acetaminophen | 70 (11.6) |
| Others | 70 (11.6) |
| Preventive treatment, n (%) | 384 (63.4) |
| Antidepressants | 114 (18.8) |
| Antiepileptic drugs | 183 (30.2) |
| Beta blockers | 48 (7.9) |
| Calcium channel blockers | 120 (19.8) |
| Others | 72 (11.9) |

**Table S3: Comparisons between patients with and without new-onset headaches after the COVID-19 pandemic**

|  | **No new-onset headache (n=511)** | **New-onset headache (n=95)** | **P value^b^** |
| --- | --- | --- | --- |
| Age, years | 45.6±12.0 | 43.2±11.7 | 0.534 |
| Diagnosis, n (%) |  |  |  |
| Migraine without aura | 415 (81.2) | 68 (71.6) | **0.032** |
| Migraine with aura | 110 (21.5) | 29 (30.5) | 0.055 |
| Chronic migraine | 69 (13.5) | 15 (15.8) | 0.554 |
| Onset of migraine, years | 20.5±10.2 | 19.0±10.2 | 0.19 |
| Smoking, n (%) |  |  | 0.905 |
| Never | 387 (75.7) | 70 (73.7) |  |
| Past | 86 (16.8) | 17 (17.9) |  |
| Current | 38 (7.4) | 8 (8.4) |  |
| Alcohol intake, n (%) |  |  | 0.711 |
| Never | 244 (47.8) | 47 (49.5) |  |
| <1 day/week | 189 (37.1) | 34 (35.8) |  |
| 1-2 days/week | 53 (10.4) | 7 (7.4) |  |
| 3-5 days/week | 13 (2.5) | 4 (4.2) |  |
| 6-7 days/week | 10 (2.0) | 3 (3.2) |  |
| Caffeine, n (%) | 475 (93.0) | 85 (89.5) | 0.239 |
| Caffeine, cups/day | 2.7±2.1 |  |  |
| Comorbidities, n (%) | 219 (42.9) | 50 (52.6) | 0.078 |
| Occupations, n (%) |  |  | 0.412 |
| Frontline worker | 158 (38.0) | 35 (36.8) |  |
| Working from home | 194 (38.0) | 30 (31.6) |  |
| Other occupation | 159 (31.1) | 30 (31.6) |  |
| Use of protective gear, n (%) | 508 (99.4) | 95 (100.0) | 0.454 |
| Mask, n (%) | 508 (99.4) | 95 (100.0) | 0.454 |
| Mask usage (h/d) | 6.6±4.0 | 7.5±4.0 | **0.041^c^** |
| Face shield, n (%) | 28 (5.5) | 9 (9.5) | 0.135 |
| Face shield usage (h/d) | 4.7±3.2 | 4.6±3.0 | 0.903^c^ |
| PPE, n (%) | 9 (1.8) | 1 (1.1) | 0.619 |
| PPE usage (h/d) | 4.0±3.2 | 3.5 | - |
| Impact of COVID-19 on daily life, n (%) | 219 (42.9) | 54 (56.8) | **0.012** |
| Problems with hospital access, n (%) | 81 (15.9) | 14 (14.7) | 0.784 |
| Shortage of medications, (n%) | 31 (6.1) | 12 (12.6) | **0.022** |
| Received online medical care | 81 (15.9) | 14 (14.7) | 0.784 |
| Interest in COVID-19, n (%) |  |  | 0.647 |
| Not at all | 1 (0.2) | 0 (0.0) |  |
| Very little | 3 (0.6) | 0 (0.0) |  |
| Little | 31 (6.1) | 6 (6.3) |  |
| Moderate | 122 (23.9) | 17 (17.9) |  |
| Strong | 354 (69.3) | 72 (75.8) |  |
| Concerns about COVID-19, n (%) |  |  | **0.004** |
| Not at all | 2 (0.4) | 2 (2.1) |  |
| Very little | 10 (2.0) | 0 (0.0) |  |
| Little | 73 (14.3) | 10 (10.5) |  |
| Moderate | 158 (30.9) | 17 (17.9) |  |
| Strong | 268 (52.4) | 66 (69.5) |  |
| Stress, n (%) |  |  | **0.012** |
| Decreased | 26 (5.1) | 4 (4.2) |  |
| Unchanged | 208 (40.7) | 24 (25.3) |  |
| Increased | 277 (54.2) | 67 (70.5) |  |
| Physical activity, n (%) |  |  | 0.175 |
| Decreased | 249 (48.7) | 56 (58.9) |  |
| Unchanged | 234 (45.8) | 34 (35.8) |  |
| Increased | 28 (5.5) | 5 (5.3) |  |
| PGIC scale (1-7) |  |  |  |
| Mood | 4.4±0.9 | 5.2±0.9 | **<0.001** |
| Sleep | 4.3±0.9 | 5.0±1.1 | **<0.001** |
| Acute headache treatment, n (%) |  |  | **<0.001** |
| Decreased | 49 (9.6) | 4 (4.2) |  |
| Unchanged | 338 (66.1) | 29 (30.5) |  |
| Increased | 124 (24.3) | 62 (65.3) |  |
| Addition or changes in preventive headache medications, n (%) | 72 (14.1) | 22 (23.2) | **0.025** |
| Pre-MIDAS total score | 13.1±20.4 | 20.6±24.6 | **<0.001^c^** |
| Pre-MIDAS A score | 23.3±20.6 | 24.5±18.9 | 0.312^c^ |
| Pre-MIDAS B score^a^ | 5.7±5.5 | 6.8±9.1 | 0.220^c^ |
| Post-MIDAS total score | 11.7±17.9 | 31.2±44.5 | **<0.001^c^** |
| Post-MIDAS A score^a^ | 22.4±20.0 | 29.0±23.8 | **0.017^c^** |
| Post-MIDAS B score^a^ | 5.6±5.1 | 7.6±9.6 | **<0.001^c^** |

PPE=personal protective equipment; MIDAS= Migraine Disability Assessment. The values in bold indicate significant differences.

a: Missing values (alcohol intake=2, caffeine=11, Pre-MIDAS B =7, mask time=22, and face shield time=1, post-MIDAS A=1, B=3) were excluded.

b: Student’s t test for continues variables or the chi-squared test for categorical variables

c: Mann-Whitney U test for non-normally distributed variables.

**Table S4: Logistic regression analysis results of new-onset headache (n=603)**

| **Variables** | **Standardized regression**  **coefficient** | **SE** | **Wald** | **OR (95% CI)** | **P value** |
| --- | --- | --- | --- | --- | --- |
| Age | -0.025 | 0.011 | 28.967 | 0.975 (0.955-0.996) | 0.017 |
| Mood | 0.874 | 0.162 | 5.571 | 2.397 (1.743-3.295) | <0.001 |
| Sleep | 0.352 | 0.894 | 50.318 | 1.421 (1.061-1.903) | 0.018 |

Using a likelihood ratio forward selection.

Independent variables included clinical variables with p<0.1 in Table S4 plus age and sex except for the total MIDAS and A and B scores and changes in acute or preventive headache treatment: age, sex, migraine with or without aura, comorbidities, mask usage (h/d), impact of COVID-19 on daily life, concerns about COVID-19, changes in stress, mood, sleep, and medication shortage.
